# Morphological evolution of silicon surfaces nanopatterned by focused ion beam irradiation

**DOI:** 10.1038/s41598-025-33947-y

**Published:** 2026-01-31

**Authors:** Dipak Bhowmik

**Affiliations:** Division Micro-robotics and Control Engineering, Department of computing Science, University of Oldenburg, 26129 Oldenburg, Germany

**Keywords:** Nanopatterning, FIB, Ripple pattern, Terrace morphology, AFM, Adhesion, Materials science, Nanoscience and technology, Physics

## Abstract

Nanopatterning on solid surface has emerged as an important tool in the field of nanotechnology, nano/micro-electromechanical systems (NEMS/MEMS), photonics, and biotechnology. Among the available techniques, focused ion beam (FIB) enables controlled and precise nanopatterning on solid surfaces. The formation and evolution of nanoripple patterns on silicon (Si) surfaces by 30 keV Ga⁺ focused ion beam (FIB) irradiation has been investigated in this study. We perform a systematic study to investigate the well-defined ripple pattern formation by conducting experiments at different ion incidence angles (0°-45°), ion energies (10–30 keV), and ion fluences. Well-defined ripple patterns are observed near 30° ion incidence angle, with ripple periodicity and amplitude strongly dependent on ion fluence. A distinct morphological transition at higher ion fluences from ripple patterns to terrace-like structures is observed. The force measurements are also carried out in terms of pull-off force test on ripple patterned Si surfaces to investigate the effect of nanopattern on adhesion force as employed by atomic force microscopy (AFM) in force mode with normal sharp probe and silica colloidal probe. The observed results reveal that one can fabricate well-defined nano ripple pattern along with terrace pattern at high fluence and adhesion force can also be tuned with nanoscale pattern. The mechanism of ripple pattern formation and its transition to terrace-like structure are discussed in the framework of well-established continuum models. Potential applications of such nanopatterned surfaces for nanoelectromechanical systems (NEMS) and other functional properties are also highlighted.

**Supplementary Information:**

The online version contains supplementary material available at 10.1038/s41598-025-33947-y.

## Introduction

The rapid advancement of nanotechnology has driven the need for precise and versatile fabrication techniques to create functional nanostructures. Ion beam is considered as a powerful and effective tool for the nanopattern formation on solid surface in a controlled and precise way^1–3^. Energetic ions can be regarded as a bunch of atoms having certain velocity. The interaction of such ions with solid surface leads to form several types of structures from micro to nanoscale depending on ion beam parameters such as ion energy, ion incidence angle, ion fluence, ion species etc. The periodic nanopattern formation on different solid surface has been studied since last few decades using low and medium energy broad ion beam irradiation^2–7^. However, the nanopatterning using focused ion beam (FIB) is now widely used as it advantages such as a high patterning rate, precise and selective etching, flexible area selection (few µm to 100 μm), thereby significantly simplifying the surface nanostructuring process^8^. Unlike electron-beam lithography, nanoimprint lithography, or photolithography, which typically require multiple processing steps such as resist coating, mask alignment, and etching, focused ion beam (FIB) irradiation enables direct, maskless surface modification. This makes FIB particularly attractive for rapid prototyping and localized nanopatterning, although each technique offers distinct advantages depending on the application. Moreover, FIB systems allow in-situ observation via scanning electron microscopy (SEM), enabling real-time control of processing parameters during nanopattern formation.

The wavelike ripple structure using ion beam is a fascinating phenomenon which has attracted the significant interest in different fields like template substrate for several deposition, anisotropy study, nanoelectromechanical systems (NEMS), controlling adhesion and friction etc^9–13^. Silicon (Si) is a keystone of modern semiconductor technology, and the nanopattern formation on it with varying dimension plays a crucial role in electronics, photonics, and energy applications. Therefore, ripple formation on Si surfaces under ion irradiation has been reported over a wide range of ion energies, incidence angles, and ion species^2,3,14,15^. The observed surface morphologies can be explained by well-established mechanism of pattern formation as first proposed by Bradley and Harper (BH)^1^ and its extensions^2,3^ through the interplay of curvature-dependent sputtering, viscous flow, and surface diffusion. The ripple periodicity, orientation, and amplitude are highly sensitive to the irradiation parameters, which essentially control the surface nanostructures. Despite extensive studies, questions remain regarding the transition between different morphological regimes at higher ion fluences.

The transformation of periodic ripple pattern to quasi-periodic terrace type of nanostructure as resembled like a sawtooth shape is an important implication of ion bombardment on solid surface especially at higher incidence angle and higher fluence^16–19^. The quasi-periodic terrace morphology is having asymmetric structure with two dominant slopes: one in front side and other in rear side, which has become interesting research for both academic purpose and potential applications in fabricating blazed gratings^20,21^. Most of the terrace formation on Si and other solid surfaces are observed at high ion fluence, at high ion incidence angle, and for some with external metal impurity deposition^22–24^. The observed morphological pattern like terrace, pyramid at higher fluence could not be explained by linear continuum model. Hence, the model was extended to explain the morphological transition at higher fluence by two-dimensional anisotropic Kuramoto - Sivashinsky (AKS) equation after adding nonlinear terms^25–27^. However, AKS equation even could not explain the morphological transition such as terrace topography at high fluence. Pearson and Bradley^20^ recently introduced a model which includes cubic nonlinear terms in AKS equation. The cubic terms in the equation of motion mainly depend on sputtering and ion implantation. Pearson and Bradley showed by numerical integration that the inclusion of cubic terms in AKS equation can generate terrace formation at higher ion incidence angle or nearly grazing incidence. Ion beam not only forms nanopattern on solid surfaces but it also modifies the interface of a material leading to changes its electrical, optical, magnetic properties^28–32^. The ion beam can also tailor the intermolecular forces^33,34^ by creating defects, sputtering, at the interface, which can further modify the adhesion properties. Nanoscale adhesion plays a crucial role in device applications particularly for NEMS/MEMS systems. However, the effect of nanopattern on adhesion has not yet been investigated.

In this study, we address the morphological evolution of Ga⁺ FIB irradiation by varying ion incidence angle, ion energy, and ion fluence. The well-defined ripple pattern formation is observed for the ion energy 30 keV at ion incidence angle 30^0^. The ripple structure was initiated from the ion fluence 3 × 10^17^ ions/cm^2^, which can be considered as threshold ion fluence for ripple pattern at this ion energy (30 keV). At higher fluence, the ripple structures become elongated along the ion beam direction and transform to step like terrace structure. The pull-off force measurements on ion-induced patterned surfaces by normal sharp probe and silica colloidal probe indicate the significant modification of adhesion force compared to its pristine surface.

## Experimental details

The present study is conducted using two important powerful technique of nanomanipulation namely focused ion beam attached with scanning electron microscopy and atomic force microscopy (AFM). Commercially available small piece (5 × 5 mm) of polished Si (100) wafers (rms roughness ~ (0.2 ± 0.03) nm) (Plano, GmbH, Germany) are cleaned in both isopropyl alcohol and acetone using an ultrasonic bath for 10 min. The cleaned Si (100) surfaces are irradiated by 10–30 keV Ga^+^ FIB using a dual-beam high-resolution scanning electron/focused ion beam microscope, namely, the Lyra FEG (TESCAN, Brno, Czech Republic). The schematic of irradiation experiment in dual beam FIB-Scanning Electron Microscope (FIB-SEM) and inside chamber image of FIB-SEM are shown in Supporting Information (Fig. S1). The constant current of 10 nA (ion dose ~ 1.56 × 10^16^ ions.cm^−2^.s^−1^) is maintained during the ion exposure for all the samples and the ion fluences are varied from 2 × 10^17^ ions/cm^2^ to 1.3 × 10^19^ ions/cm^2^ for 30 keV ion energy. Before each irradiation experiment, the ion beam current was carefully adjusted to approximately 10 nA and allowed to stabilize. After completing each irradiation run, the beam current was measured again, and we consistently observed only very small variations. Such minor fluctuations introduce only minimal uncertainty in the fluence calculation and do not affect the overall nanopatterning outcome. The beam spot size of 4000 nm with 90% overlap and dwell time of 0.8 µs was fixed and 30 × 30 µm^2^ area was selected for the ion beam irradiation. The scan speed determined the pixel dwell time, and together with the beam spot size and pixel spacing, this produced significant beam overlap. Under these conditions, the scanned region experienced quasi-homogeneous ion irradiation. The irradiation was also performed using smaller areas and lower beam currents. Although nanopatterns could still be generated under those conditions, the sputtering became highly localized within the small scan region, causing rapid pit formation and strong curvature-dependent sputtering near the edges. These effects disrupted the uniform evolution of surface morphology and made it difficult to observe ripple development over a broad fluence range. In contrast, irradiation over a larger 30 × 30 μm² area distributes the sputtering more uniformly, minimizes edge-related curvature effects, and maintains a sufficiently flat central region throughout the irradiation process. This allows the ripple morphology to evolve consistently from the linear to the nonlinear regime. For this reason, we selected an optimum irradiation area of 30 × 30 µm^2^ and a beam current of 10 nA, which ensured stable sputtering conditions and allowed consistent comparison of ripple morphologies across all fluences. The irradiation was performed in vacuum with chamber pressure < 10^− 2^ Pa and FIB Gun pressure around 7 × 10^− 6^ Pa. The images were taken by SEM after each ion bombardment and pattern formation was confirmed.

### AFM tests and analysis

The irradiated Si samples are further characterized by inhouse AFM (NanoWizard II setup, Bruker Nano GmbH, Germany) for high-resolution topography. The morphologies of ion bombarded Si surfaces are captured in intermittent contact (IC) mode in air by sharp AFM probe (Budget Sensors, Tap 300-G) with tip apex radius 10 nm. The pull-off force tests are conducted by both specially fabricated silica colloidal probe having radius (520 ± 50) nm and commercial sharp probe (Force modulation Multi75GD-G, Budget Sensor Probe) having tip apex radius ~ (30 ± 10) nm. The adhesion map has been conducted by capturing 32 × 32 force map with maximum normal force 20 nN and vertical tip velocity 1 μm s^-1^. The force constant of colloidal probe and sharp probe are 0.39 N/m and 1.75 N/m, respectively as calculated from the calibration. The method of colloidal probe fabrication by robotic nanomanipulation inside SEM chamber is already described in our earlier works^35–37^. The SEM images of colloidal probe and sharp AFM probe are provided in Supporting Information (Fig. S4). The captured images are processed and further analyzed for root means square (rms) roughness, ripple wavelength, amplitude, etc. from WSxM software^38^ followed by parabolic flattening of the image. The slope angle distribution is extracted from Gwyddion software^39^. The ion penetration depth is estimated using Transport of Ions in Matter (TRIM) simulation^40^ (See Fig. S5 in Supporting Information).

## Results and discussions

### Morphological study

**Fig. 1:**
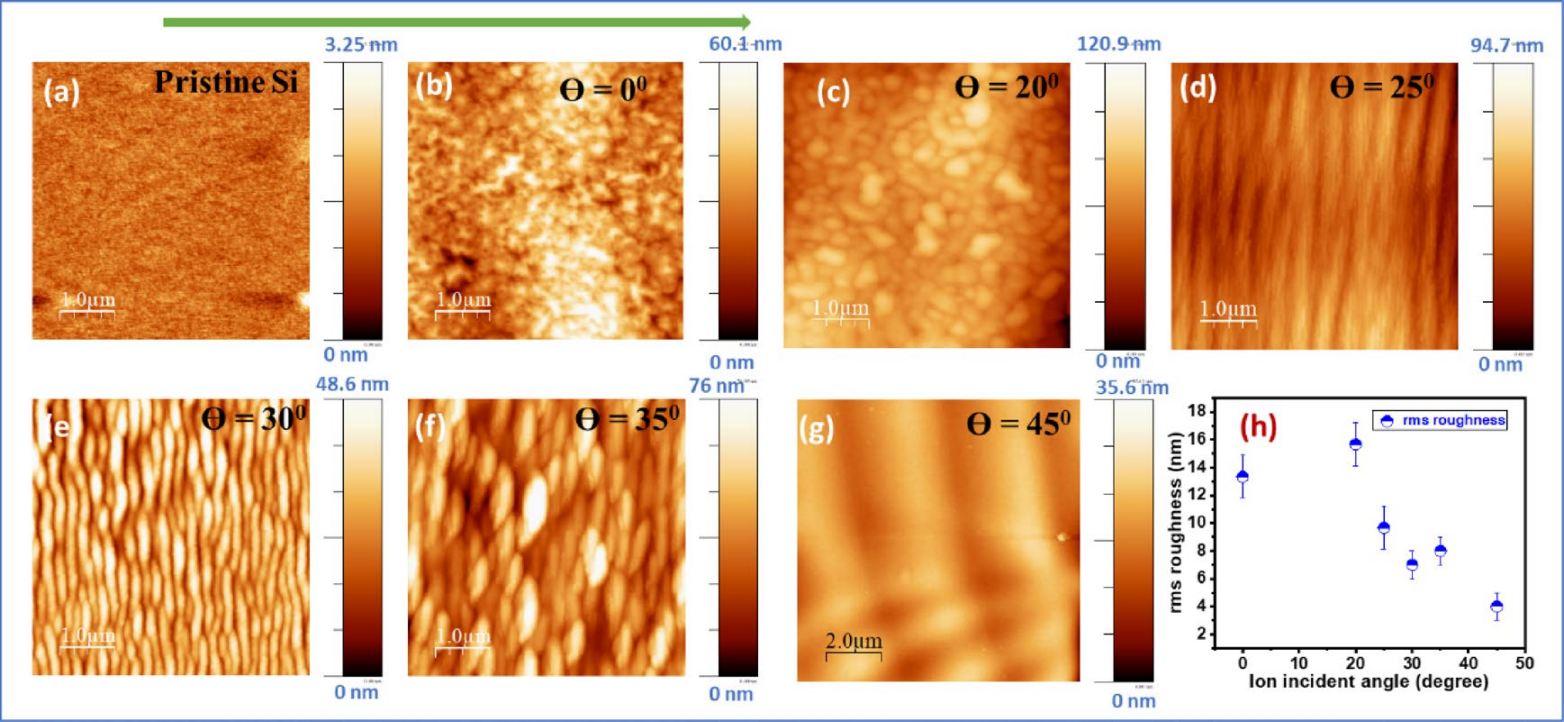
AFM images of (**a**) pristine Si, (**b**–**g**) 30 keV Ga^+^ bombarded Si with different ion incident angles ($\:\theta\:$) as indicated on images with constant ion fluence 1 × 10^18^ ions/cm^2^. (**h**) RMS roughness of ion bombarded Si surfaces by 30 keV Ga bombardment at different angles. The arrow on the top indicates ion beam direction.

Figure 1 presents atomic force microscopy (AFM) images of pristine Si and 30 keV Ga^+^ FIB irradiated Si surfaces at different ion incidence angles with constant ion fluence (1 × 10^18^ ions/cm^2^). The pristine Si surface as shown in Fig. 1 (a) displays smooth and atomically flat surface with minimal roughness (~ 0.2 nm). Upon irradiation by 30 keV FIB at normal incidence irradiation ($\:\theta\:$= 0°), the sputtering and ion deposition on Si surface develops irregular granular type of features, resembling flower-like structures. A similar type of morphology is observed at $\:\theta\:$ = 20°, although the structures appear more compact. With increasing incidence angle to $\:\theta\:$ = 25°, an elongated surface feature begins to align along the ion beam direction. A well-defined wavelike ripple pattern emerges distinctly at $\:\theta\:$ = 30°. When the incidence angle is increased to $\:\theta\:$ = 35°, the ordering of ripples weakens, resulting in irregular and coarser surface features. However, no such ripple pattern is observed at $\:\theta\:$ = 45° and above, leaving the surface with broad undulations. The root-means-square (rms) roughness as depicted in Fig. 1 (h), is highest at $\:\theta\:$ = 0° and 20°, where random sputtering and cluster-like growth generate irregular height fluctuations. At $\:\theta\:$ = 30°, the surface reorganizes into ordered ripples, which reduce stochastic variations and thus lower the roughness despite strong anisotropy. Beyond 35°, the ripple order deteriorates into broad undulations, but the roughness remains lower than at normal incidence, reflecting the increasing role of surface relaxation at higher angles. The ion bombardment was also conducted on Si surface by 10 keV, 15 keV, 20 keV, and 25 keV Ga^+^ FIB at an ion incidence angle of 30^0^. The AFM morphologies of those ion bombarded Si surfaces are shown in Supporting Information (Fig. S3). It is observed that the nano ripple patterns are not well-ordered and having low amplitude even after the ion bombardment at ion fluence 1 × 10^18^ ions/cm^2^. This may be due to insufficient instability generation at this ion energy and fluence.

 To avoid any edge-related artefacts, all AFM analyses in this study were carried out at the center of the irradiated region, where the ion flux and sputtering conditions are most uniform. At the perimeter of the scanned area, the sputtering rate can vary due to beam-profile and partial scan overlap of the rastered beam, which may lead to non-uniform material removal. Although the edge region indeed shows measurable sputtering depth, the central area provides a far more reliable representation of the intrinsic nanopattern evolution. The irradiation at a relatively large 30 μm×30 μm area for each fluence is expected less sputtering depth.

**Fig. 2:**
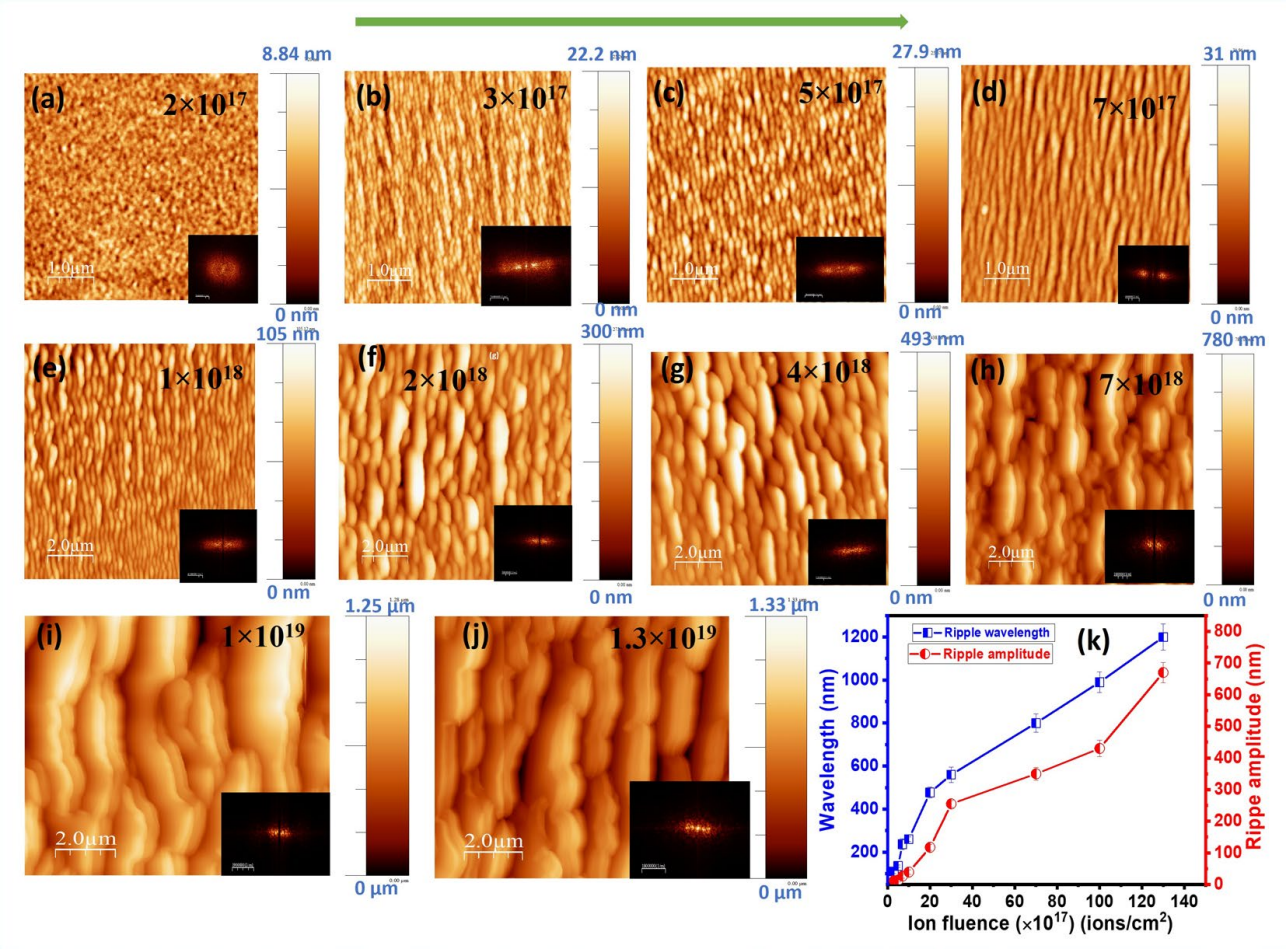
(**a**–**j**) AFM images of 30 keV Ga^+^ bombarded Si with different ion fluences as written on the images with constant ion incidence angle 30^0^. The scan area for (**a**–**d**) is taken in 5 × 5 µm^2^ area and for (**e**–**j**) 10 × 10 µm^2^ area is taken for the large structure. FFT images are shown at the right corner of each AFM images, indicating the ripple direction for the patterned surfaces. The arrow on the top indicates ion beam direction. (**k**) Variation of ripple wavelength and ripple amplitude with ion fluence.

To study the morphological evolution of ripple structure, we have extended the ion irradiation by 30 keV Ga^+^ ion on Si surface at different ion fluences with constant ion incidence angle 30^0^. The 2-D AFM images of 30 keV Ga^+^ bombarded Si surfaces at constant ion incidence angle 30^0^ with varying ion fluences are shown in Fig. 2 (a-j) indicating the fluence dependent morphology. The ripple wavelength and amplitude as calculated from the AFM images (See S2 in Supporting Information) are plotted with ion fluence in Fig. 2 (k). At a low fluence of 2 × 10^17^ ions/cm^2^, the surface remains relatively featureless with only short-range irregularities. Quasi periodic variation of height amplitude resulting in ripple structure was observed upon increasing the fluence to 3 × 10^17^ ions/cm^2^. The ripple pattern becomes more distinct and periodic with further increase of ion fluence to 5 × 10^17^ and 7 × 10^17^ ions/cm^2^. The wavevector of ripple structures are developed along the ion beam direction. At higher fluences (1 × 10^18^ to 7 × 10^18^ ions/cm^2^), the ripple morphology coarsens, with both the wavelength and amplitude increasing, as confirmed by the quantitative analysis in Fig. 2 (k). Beyond 1 × 10^19^ ions/cm^2^, the ordered ripples transform into terrace-like structures with broader, step-like modulations, indicating a transition from well-ordered periodic ripples to large-scale surface faceting. The corresponding fast Fourier transform (FFT) images are also shown at the right corner of each AFM images, which indicates the ripple direction and evolution. Sharp spots at intermediate ion fluences reflect strong ripple ordering, while diffused patterns at the highest fluences indicate loss of periodicity. The plot in 2 Fig. (k) quantifies this behavior: both ripple wavelength and amplitude increase monotonically with ion fluence, consistent with the coarsening dynamics of ion-induced surface patterns. This is called roughening of the ripple and it is obvious most of the time due to ion induced sputtering effect^22,41^. It is interesting that with increasing ion fluence the ripple amplitude is close to the ripple wavelength. This phenomenon indicates a nonlinear surface instability where surface undulations grow significantly due to competing physical mechanisms such as ion sputtering, stress relaxation, or material mass redistribution. This type of phenomena was also observed earlier by other groups at higher ion fluences^22,42,43^. At very high fluences, however, the transition to terrace-like morphology suggests that sputtering and mass redistribution processes dominate over the curvature-dependent instability that drives ripple formation. To go further insight of the morphological evolution induced by ion irradiation, statistical parameters like rms roughness and correlation length are extracted from the AFM images as will be discussed below.

**Fig. 3:**
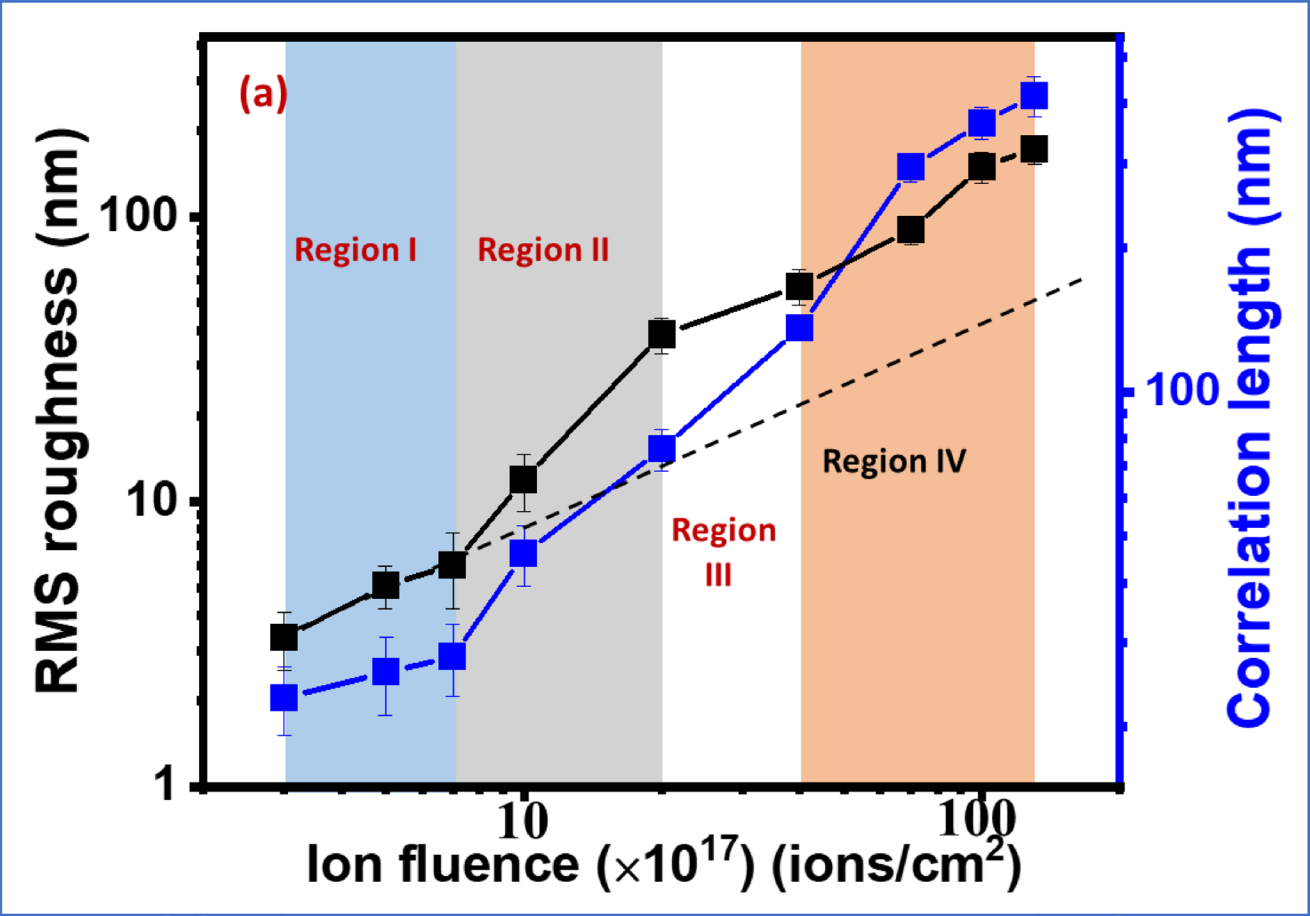
(**a**) RMS roughness and correlation length along the ripple direction as a function of ion fluence. The black curve represents the RMS roughness and blue curve represents the correlation length. The plot uses a logarithmic scale for comparison of the RMS roughness and correlation length. The black dashed line represents a linear reference, highlighting the approximately linear increase of RMS roughness at lower ion fluences and its deviation from linear behavior at higher fluences.

The rms roughness and correlation length for each patterned surface are calculated and plotted in Fig. 3 (a). The correlation length is a statistical measure of how far the surface features remain correlated (proportional to lateral dimension) and rms roughness indicates the variation of vertical amplitude^44^. These two parameters describe the vertical and lateral dimensions of the surface morphology, analogous to ripple amplitude and wavelength, but are obtained from AFM images through different statistical analyses and are therefore not same. The morphological evolution of ripple pattern can be understood from this plot and we divide the variation in 4 different regions as indicated in the figure. Initially, in region I, the rms roughness (i.e., amplitude) increases slowly, whereas the correlation length remains almost constant. Here, the RMS roughness increases linearly as given in the reference line (dashed black) in Fig. 3. As the ion fluence increases (region II), both the rms roughness and the correlation length grow significantly. The non-linearity becomes important in this region where the vertical amplitude grows faster than the correlation length. The RMS roughness in this region starts to deviate from the linear reference line as indicated in Fig. 3. Here, the nonlinearity refers to the nonlinear evolution of the surface height as a function of ion fluence (irradiation time). While the initial growth of surface height follows the linear Bradley-Harper regime, higher-order nonlinear terms become dominant at larger ion fluences, leading to coarsening, and morphology transitions. The increasing roughness indicates the amplification of surface undulations as also reflected in the ripple amplitude (Fig. 2k). In region III, the amplitude and correlation length increase slowly, reflecting the establishment of a steady-state ripple wavelength. Finally, in Region IV, a further increase in both roughness and correlation length is observed. In this regime, surface smoothing mechanisms compete with roughening, leading to the formation of large-scale, spatially coherent ripple domains without a proportionally large increase in height (roughness). This behavior indicates the onset of a nonlinear morphological transition, in which higher-order terms in the continuum description become dominant.

**Fig. 4:**
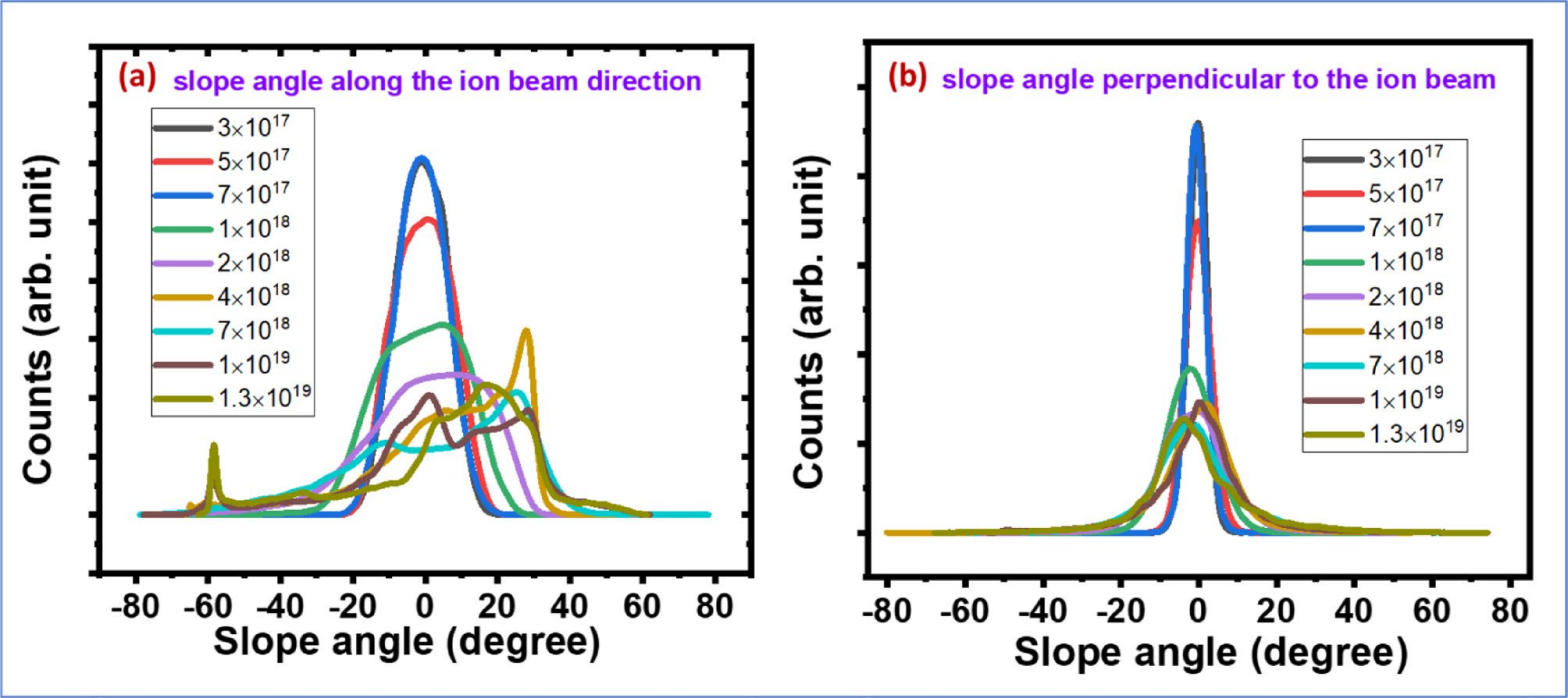
(**a**) One-dimensional slope ($\:\partial\:h/\partial\:x$) angle distribution along the ion beam direction and (**b**) slope ($\:\partial\:h/\partial\:y$) angle distribution perpendicular to the ion beam direction for nanopatterned surfaces.

**Fig. 5:**
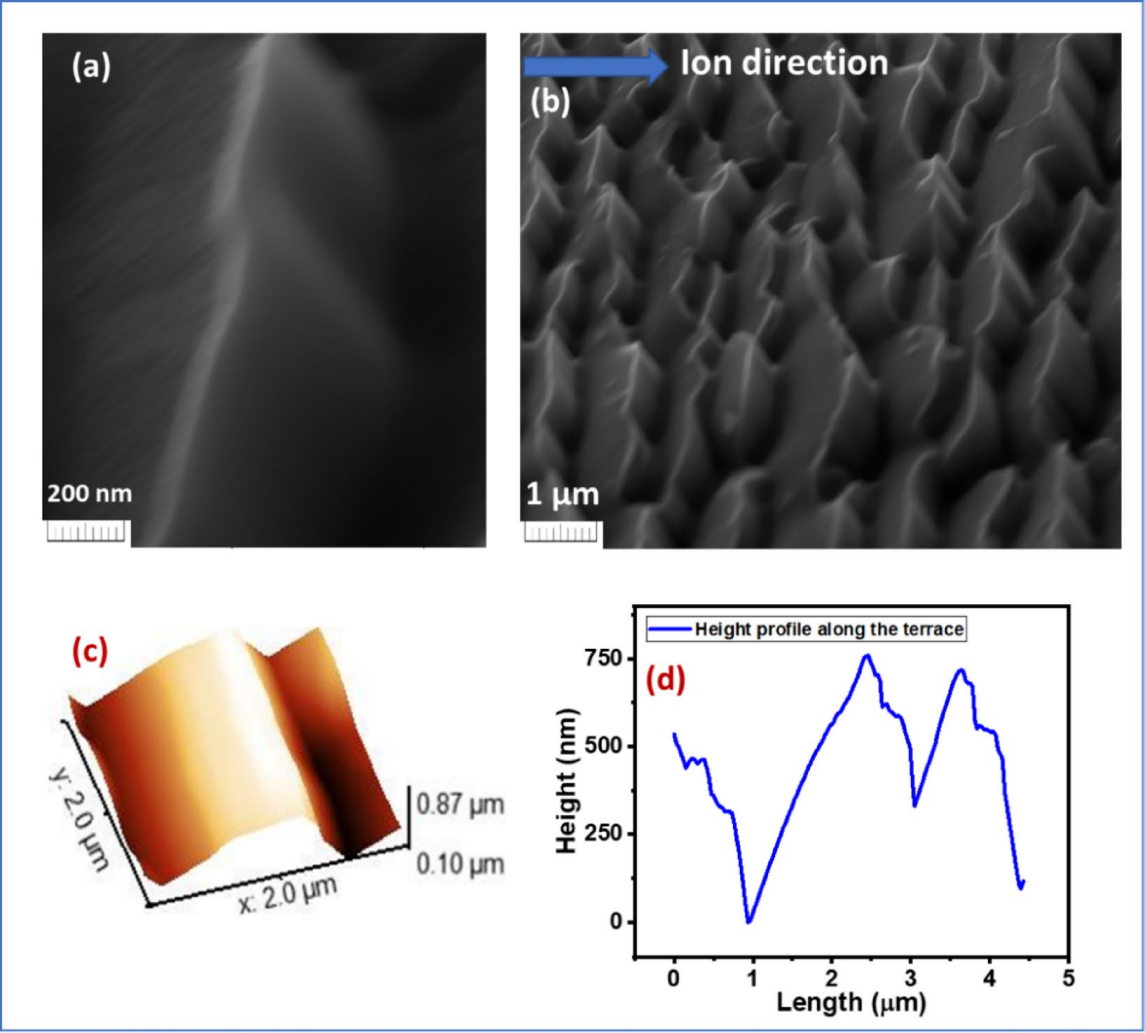
(**a**) and (**b**) Cross-sectional scanning electron microscopy (SEM) view of terrace structure for 30 keV Ga^+^ ion bombardment on Si surface with ion fluence 1** × **10^19^ ion/cm^2^. (**c**) 3-dimensional AFM image of terrace morphology as observed at high fluence (1 × 10^19^ ion/cm^2^). (**d**) The height profile along the terrace structure.

The morphological evolution of ion-irradiated Si surfaces can further be understood from the distribution of local slope angles as extracted from AFM topography data. Figure 4 (a) and (b) show one-dimensional slope angle distribution along the ion beam direction and perpendicular to the ion beam direction, respectively. At lower ion fluences (e.g., 3 × 10^17^ to 7 × 10^17^ ions/cm^2^), the slope distributions along the ion beam direction exhibit a narrow symmetric peak, indicating the formation of regular, sinusoidal ripple structures. This is characteristic of the early ripple regime, where surface height modulations are small and periodic, consistent with the predictions of linear Bradley-Harper (BH) theory. As the ion fluence increases to intermediate values (1 × 10^18^ to 4 × 10^18^ ions/cm^2^), the slope distributions broaden and become asymmetric, with a shift of the peak at higher angles. This broadening may be the indication of an increase in surface steepness and asymmetry in ripple profiles, suggesting the beginning of nonlinear effects. At higher fluence (7 × 10^18^ to 1.3 × 10^19^ ions/cm^2^), the slope angle distributions exhibit multiple peaks with more intense peak at the front side of ion beam incidence. The negative slope at rear side of ion incidence leads to the steeper height variation as also shown in Fig. 5 (d). On the other hand, the slope distribution along the perpendicular direction of ion beam remains symmetric, but broadens the peak at high fluence along with slight peak shift for some ion fluence. This also indicates a transition from a well-ordered ripple morphology to a more disordered, complex terrace like surface topography due to high ion fluence.

For a clearer understanding of the terrace morphology, representative cross-sectional SEM view and 3-dimensioanl AFM images were acquired at different scales for 30 keV Ga^+^ ion bombardment on Si surface at ion fluence 1** × **10^19^ ion/cm^2^ as shown in Fig. 5. The cross-sectional view of terrace morphology has been taken by tilting the stage which makes an angle (60^0^) with the electron beam. The cross-sectional SEM images (Fig. 5a and b) highlight the steep step-like terrace structures, aligned with respect to the ion beam direction. The AFM measurements (Fig. 5c) provide a three-dimensional view of the terrace topography, and the corresponding height profile (Fig. 5d) quantitatively confirms the step-like features along with large amplitude modulation of the surface. Together, these characterizations give us a clear visualization of the terrace evolution, thereby supporting the proposed mechanism of ripple transition into faceted terrace-like morphologies under high ion fluence.

## Adhesion force measurement

**Fig. 6:**
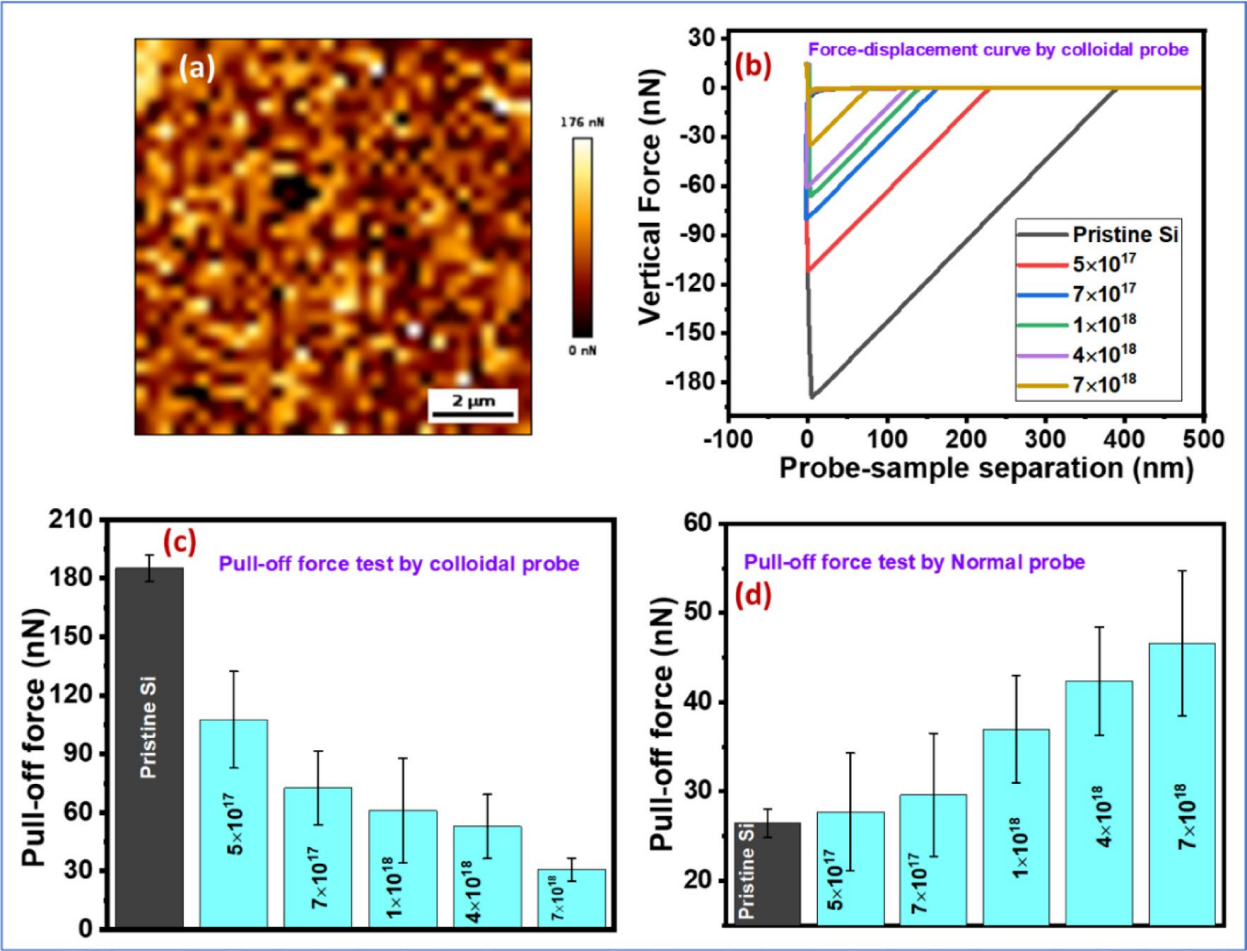
(**a**) Pull-off force map by silica colloidal probe on ripple patterned Si surface as irradiated with ion fluence 5 × 10^17^ ion/cm^2^. (**b**) Force-distance curve conducted by colloidal probe AFM on pristine Si and ion bombarded Si surfaces. (**c**) and (**d**) Average pull-off force on pristine Si and ion bombarded Si surfaces as measured by colloidal probe and normal sharp commercial probe, respectively. The standard deviation in pull-off force comes from the average of 30 force-distance curves for each probe-substrate combination.

The ion beam not only creates nanopattern on solid surface but it also modifies intermolecular forces. Adhesion measurements are carried out on pristine and ripple-patterned Si surfaces to investigate the effect of nanopatterning on its adhesion force. The pull-off force map on pristine and ion-bombarded Si surfaces by silica colloidal probe was conducted as presented in Fig. 6 (a). Typical force-distance curve on pristine and ion-bombarded Si surfaces measured by colloidal probe is also shown in Fig. 6 (b). The average pull-off force is calculated from 30 individual force-distance curve on each surface and given in Fig. 6 (c) and (d) by employing both colloidal probe and normal commercial sharp AFM tip. The pull-off force, which is a measure of force to detach the probe from the surface, measured by silica colloidal probe (radius ~ 520 ± 50 nm) is found to decrease for patterned surfaces from its pristine surface. Interestingly, the pull-off force measured by normal sharp AFM tip (~ tip radius 30 ± 10 nm) is found to increase for nanopatterned surface from its pristine surface. The interaction area for colloidal probe with smooth (pristine) Si surface is much more than for sharp tip leading to more pull-off force for colloidal probe. However, for nanopatterned surfaces, the surface is rough with periodic modulations and there are multiple peaks on the surface. Hence, the interaction area of colloidal probe and patterned surfaces is much less than its flat surface and it decreases with ion fluence due to increase of surface roughness and ripple amplitude. In case of sharp commercial probe having tip apex radius much smaller than the wavelength of the ripple structure faces multiple asperities on the surface enhancing the effective contact area between the tip and surface. The adhesion energy can easily be calculated from the pull-off force measurements as already reported in our earlier work^36,45^. However, the determination of adhesion energy from pull-off force for rough surfaces are not straightforward as the surface is not smooth and there are multiple asperities^46,47^. The ability to tailor adhesion through controlled ion-beam nanopatterning suggests for further development towards technological applications such as nanoelectromechanical systems (NEMS), where the adhesion can govern the movement of nanoscale components without stiction and wearing out.

## Discussions

The wavelike ripple structure is developed on smooth and flat solid surface after continuous ion bombardment due to the generation of instabilities on the surface. These instabilities may come from various sources such as initial perturbation, external impurities, presence of beam impurities, chemical reaction with the surface etc^41,42,48–51^. The energetic ions transfer its energy and momentum to the corresponding atoms in the surface during the interaction, which leads to sputtering and mass-redistribution. The ripple pattern formation on Si surface is generally formed at this ion energy (10’s keV) around oblique incidence angle 60^0^ and the chemical phase of Si surface also changes for reactive like ion bombardment (N_2_^+^, C^+^, O^+^)^5,41,43,48,49^. However, the ripple pattern for 30 keV Ga^+^ FIB is formed at ion incidence angle 30^0^. The ripple structures that develop on Si at an ion incidence angle 30^0^ in this study show clear agreement with the earlier observations reported by 30 keV Ga^+^ focused ion beams^52,53^. Habenicht et al.^52^ demonstrated that Ga^+^ ion sputtering can generate self-organized ripple patterns on Si surface, and that ripple propagation and wavelength evolution arise from curvature dependent sputtering under oblique incidence. Smirnova et al.^53^ reported that periodic ripples form on Si under 30 keV Ga irradiation within a similar angular window and that ripple nucleation begins once the fluence exceeds approximately 2 × 10¹⁷ cm⁻². Their results also showed that the ripple orientation is perpendicular to the beam projection, consistent with our observations. This comparison confirms that the initial ripple formation mechanism in our experiments is driven by the same sputtering induced instabilities described in the earlier focused ion beam studies, while extended irradiation leads to the development of asymmetric terrace. The topographic inhomogeneity on the ripple nucleation process has been demonstrated experimentally by several researchers. For example, Karmarkar et al.,^49^ demonstrated that the initial surface roughness of the Si surface, induced by a preliminary chemical treatment using 16.7 keV O_2_^+^ ions, reduces the ion fluence required for relief nucleation on the Si surface by two orders of magnitude. There are many reports where the relief nucleation for ripple pattern was generated from the simultaneous sputtering of inert ions and depositions of metal atoms^50,51^. We also observed the pattern formation by chemical compound formation on Si surface by beam impurity and 14 keV chemically active compound NO^+^ ion bombardment^41^. The reactive ions can create the chemical compounds like silicon nitride, carbide, and oxynitride during ion bombardment and generates surface instability due to the unequal sputtering of Si and its compounds for ripple pattern formation at ion fluence around 2 × 10^17^ ions/cm^2^. The preferential sputtering of multielement surface like muscovite mica by 12 keV inert and reactive ions generates instability for the ripple pattern formation^42^. The inert ion can only create ripple pattern on Si surface by 10’s keV energy at much higher ion fluence (~ 6 × 10^19^ ions/cm^2^)^54^.

It was observed experimentally by cross-sectional transmission electron spectroscopy (TEM) that Ga atoms in the near-surface layer of Si for 30 keV Ga^+^ ion bombardment at normal incidence with ion fluence 10^17^ ions/cm^2^ are present as precipitates dissolved within this layer, forming two distinct layers of nanoscale clusters at a depth of 10–20 nm^55,56^. This indicates that relief nucleation at incidence angles near the surface normal begins at depths corresponding to the location of Ga precipitates within the near-surface layer of Si. In a recent study of 30 keV Ga^+^ ion bombardment on Si surface, Bachurin et al.,^57^ obtained the Ga concentration near the surface by secondary ion mass spectrometry (SIMS) analysis and on the surface by Auger electron spectroscopy (AES). It was observed that the Ga concentration on the surface is about 30% for the ion incidence angle 0-30^0^ and after that the concentration of Ga decreases sharply, which may be the reason for the absence of well-defined ripple pattern beyond 30^0^ ion incidences. For present experimental condition, the ion can penetrate maximum 24 nm inside the Si surface as calculated from TRIM simulation (See Fig. S5 in Supporting Information for ion distribution profile). The surface binding energy of Ga and Si is not same, hence the sputtering yield of Si and precipitated Ga in the near surface is different, which causes the surface instability for the ripple pattern formation. The transformation of ripple to terrace morphology at higher ion fluence is consistent with the linear continuum model at the initial stage, and is further described by the newly developed model proposed by Pearson and Bradley^20^. It was also observed experimentally that the terrace or such type of topography was formed at higher ion incidence angle^22–24^. However, in the present case, the terrace topography is formed at ion incidence angle of 30^0^, which may be interesting for the community to extend the continuum model for explaining this type of topography. Hans et al., also observed the terrace morphology on glass surface by low energy Ar^+^ ion bombardment at ion incidence angle around 45^0^^58^.

**Fig. 7:**
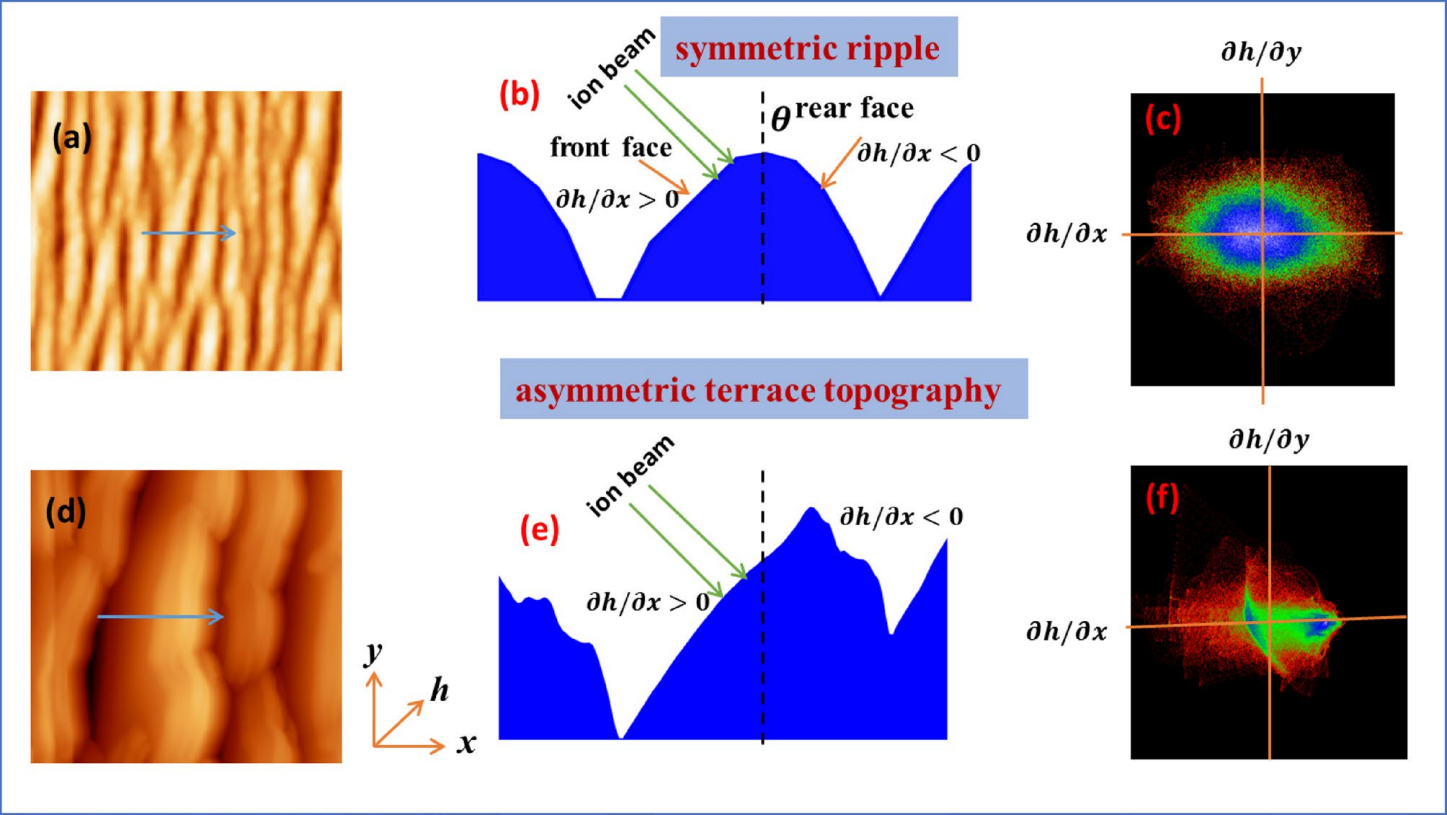
Schematic of morphological transition from ripple to terrace structure illustrating the 2D slope distributions. AFM images in (**a**) and (**d**) are taken from Fig. 2 (**d**) and (**j**). The middle line profile (**b**,**e**) represents the height profile along the marked line of (**a**) and (**d**). The slope distributions for ripple and terrace structure are shown on the right in (**c**,**f**).

The morphological evolution of ripple pattern and transforming to terrace type of topography is schematically portrayed in Fig. 7. The representative AFM images of symmetric ripple pattern by 30 keV Ga^+^ ion on Si surface (7 × 10^17^ ions/cm^2^) and terrace structure as bombarded with same ion energy with higher ion fluence (1.3 × 10^19^ ions/cm^2^) are given in the schematic along with their height profiles (middle images). The 2D slope distribution are also shown on the right images, which indicate that the front side and rear side slope are symmetric for ripple structure, however, these are not symmetric for terrace structure as also reflected in 1D slope angle distribution (Fig. 4a & b). The long-time ion bombardment makes the front side steeper due to cumulative effect of preferential sputtering, mass redistribution, and ion implantation.

The overall investigation demonstrates that FIB can give a highly controllable route to engineer nanoscale surface morphologies on Si surface through a morphological evolution from smooth surface to ripple and terrace-like structures. While FIB dwell-time modulation can produce periodic features with high geometric control, it cannot provide the physical instability mechanisms responsible for ion-induced pattern formation. The spontaneous ripples and terraces observed here arise directly from sputter-erosion dynamics and surface-diffusion effects, offering an experimental validation of Bradley-Harper and other models as discussed in this paper. The angular dependence, characteristic wavelengths, and morphology transitions obtained here are consistent with these theoretical predictions, highlighting the relevance of spontaneous pattern formation for understanding fundamental ion-surface interactions. The present study of periodic nano ripple pattern formation on Si surface with varying dimension could be potentially useful for several applications such as template for the growth of nano wire, dot, thin films; sensor for NEMS/MEMS device; optical devices like antireflective coating surface. Moreover, the adhesion measurements confirm that ion-induced nanopatterning can effectively tailor interfacial forces, which could be useful for potential applications in micro- and nanoelectromechanical systems where surface interactions are very crucial. The ion beam not only creates the periodic nanopattern formation on the surface but also modifies its optical, electrical, and mechanical properties^28–30,59,60^. We reported earlier that the optical absorption was enhanced on rippled Si surfaces due to increased surface area^59^, which is useful for solar device. FIB enables the controlled fabrication of nanopatterns on micron-scale areas, which is particularly useful for prototyping and for applications that require localized surface modification, such as specific components in NEMS/MEMS devices.

## Conclusion

The present work highlights well-defined periodic ripple pattern at nano scale on Si surface and their morphological evolution by 30 keV Ga^+^ ion generated from FIB at oblique ion incidence. A well-defined ripple pattern develops at an ion incidence angle of 30^0^ with the surface normal, at an ion energy of 30 keV, and a threshold ion fluence of 3 × 10^17^ ions/cm^2^. The precipitation of Ga atoms in the surface during ion bombardment may contribute to surface instability through the unequal sputtering of Si and implanted Ga, which in turn leads to ripple formation after sufficient irradiation. The morphological evolution in terms of ion fluence shows that ripple structure becomes elongated and it transforms to step like terrace morphology at higher ion fluence. The periodic ripple pattern is explained by linear and non-linear extensions of BH theory and the transition of terrace morphology at higher fluence is elucidated by adding cubic nonlinear terms in AKS equation as proposed by Pearson and Bradley. Keeping in mind the technological importance of nanopatterned surfaces such as for NEMS/MEMS device systems, the adhesion measurement on patterned Si surfaces indicates that the adhesion force can be well tuned with nanopatterning. However, the exact adhesion energy calculation for rough surface from pull-off force measurement needs more advancement of theoretical understanding. Additionally, detailed tribological studies on nanopatterned surface such as lateral friction, elastic modulus could potentially be useful for technological importance. Therefore, future work should be focused on friction measurement and advance model for adhesion energy calculation from pull-off force for rough surface. Despite some technological challenges and limitation on nanopatterning by FIB such as beam broadening, surface charging mainly for insulating material, FIB irradiation offers a straightforward and maskless approach for generating nanopatterns over extended areas, demonstrating its potential for controlled surface modification. We believe that the present studies not only deepen the fundamental understanding of ion-solid interactions but also cover the way for the design of functional surfaces with tunable mechanical and morphological properties.

## Supplementary Information

Below is the link to the electronic supplementary material.

Supplementary Material 1

## Data Availability

The data that support the findings of this study are available from the corresponding author upon reasonable request.
