# Supplementary material for: Morphological evolution of silicon surfaces nanopatterned by focused ion beam irradiation
Source: Sci Rep. 2026 Jan 31;16:4308. doi: 10.1038/s41598-025-33947-y (PMC12860792; doi:10.1038/s41598-025-33947-y)
Supplement: Supplementary file 1 — Supplementary Material 1 [file 41598_2025_33947_MOESM1_ESM.docx]

**Supporting Information**

**Morphological Evolution of Silicon Surfaces Nanopatterned by Focused Ion Beam Irradiation**

*Dipak Bhowmik^*^*

Division Micro-robotics and Control Engineering, Department of computing Science, University of Oldenburg, D-26129 Oldenburg, Germany

*Corresponding author email: [dipak.bhowmik@uni-oldenburg.de](mailto:dipak.bhowmik@uni-oldenburg.de)

**S1. Schematic of Experimental Set up**

The schematic diagram of experimental set up and photograph of inside chamber are shown in Fig. S1. Focused Ion Beam-Scanning Electron Microscope (TESCAN FIB-SEM) dual-beam system integrates the FIB and SEM at a specific angle within a single setup, allowing precise sample positioning at a common focal height for seamless imaging and processing. The FIB and the SEM make an angle 55^0^ with each other. The sample stage is below the SEM making perpendicular with the placed sample and SEM. The sample stage can be titled, which can change the angle between SEM and sample. Without any tilt of the sample stage, the FIB makes an angle 55^0^ with the sample. Hence, suitable angle can be adjusted during the FIB irradiation by tilting the sample stage. For imaging and FIB irradiation, the standard distance 9 mm between sample and FIB/SEM is maintained. A liquid metal ion source (LMIS), usually gallium
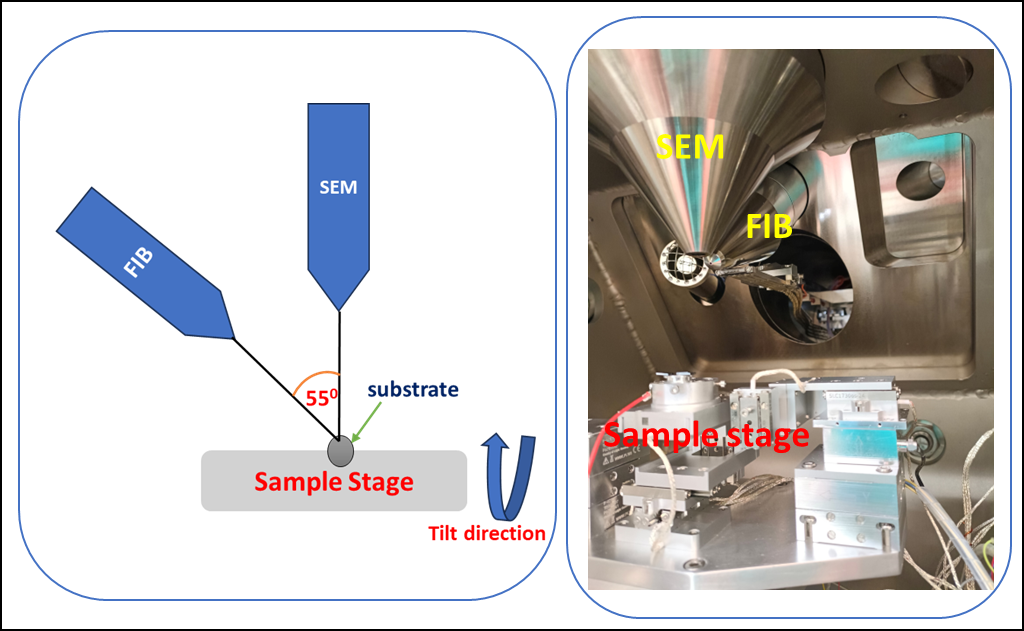
(Ga⁺) due to its stable emission properties, is used in FIB system.

**Figure S1**: Schematic of ion irradiation experiment by dual beam TESCAN FIB/SEM set up and photograph of vacuum chamber where the ion bombardment is executed.

**S2. Analysis of Nanopattern**

Fig. S2 illustrates the AFM, SEM images, height profile, auto-correlation function, and power spectral density of 30 keV Ga^+^ FIB irradiation on Si surface with 30^0^ ion incidence angle and with constant ion fluence of 7×10^17^ ions/cm^2^. Two-dimensional and three-dimensional AFM images of ion irradiated Si surface are shown in Fig. 2(a) and (b), which indicate the periodic ripple pattern formation on Si surface by 30 keV Ga^+^ FIB irradiation at oblique angle incidence (30^0^). The height profile of along the ripple structure (marked green line on Fig. 2 (a)) is plotted in Fig. 2 (c), which shows the periodic variation of height along with their amplitude and separation. The ripple amplitude is calculated from the average height of this profile for each fluence (Fig. 2 k). The Fast Fourier Transform (FFT) image and 2-D auto-correlation image are shown in the inset images on the right corner of Fig. 2 (a). The FFT indicates the ripple wavevector direction along the ion beam in the present case and the 2D-auto-correlation image measures the similarity of a surface feature at different spatial positions. The auto-correlation image also indicates the periodicity of the structure. The bright spot in the center elucidates the periodicity of the structure and the line profile along this center is auto-correlation function (ACF) as shown in Fig. S2 (e). ACF also indicates the periodic feature of the structure and the wavelength can be calculated from the central peak and first co-relation maximum (Fig. S2 (e)). The ACF is defined as [1]

$C\left( r_{1},r_{2} \right)=\sum Z(x,y)Z(x+r_{1},y+r_{2})$ (S1)

$Z(x,y)$ is the image matrix and the equation representing an image at position ($x,y$) and ($x+r_{1},y+r_{2}$) i.e., a shifted image at ($r_{1},r_{2}$). $C\left( r_{1},r_{2} \right)$ defines the similarity of the two images. The higher the similarity between the two images, the greater the autocorrelation value. The periodic pattern in the autocorrelation image is a sign of periodic pattern in the original image. Fig. S2 (d) shows the SEM image of ripple pattern at large scale captured in-situ just after the ion irradiation. This is used as a preliminary confirmation of ripple pattern formation after the ion exposure in the SEM chamber. The power spectral density (PSD) highlights periodic surface features that may seem random and visually represents their distribution across different spatial frequencies. PSD is computed from the Fourier transform of the auto-correlation function. PSD function is extracted from Fig. S2 (a) and is depicted in Fig. S2 (f). The peak in PSD also indicates the periodic nature of the structure and this corresponds to the wavelength of the ripple.


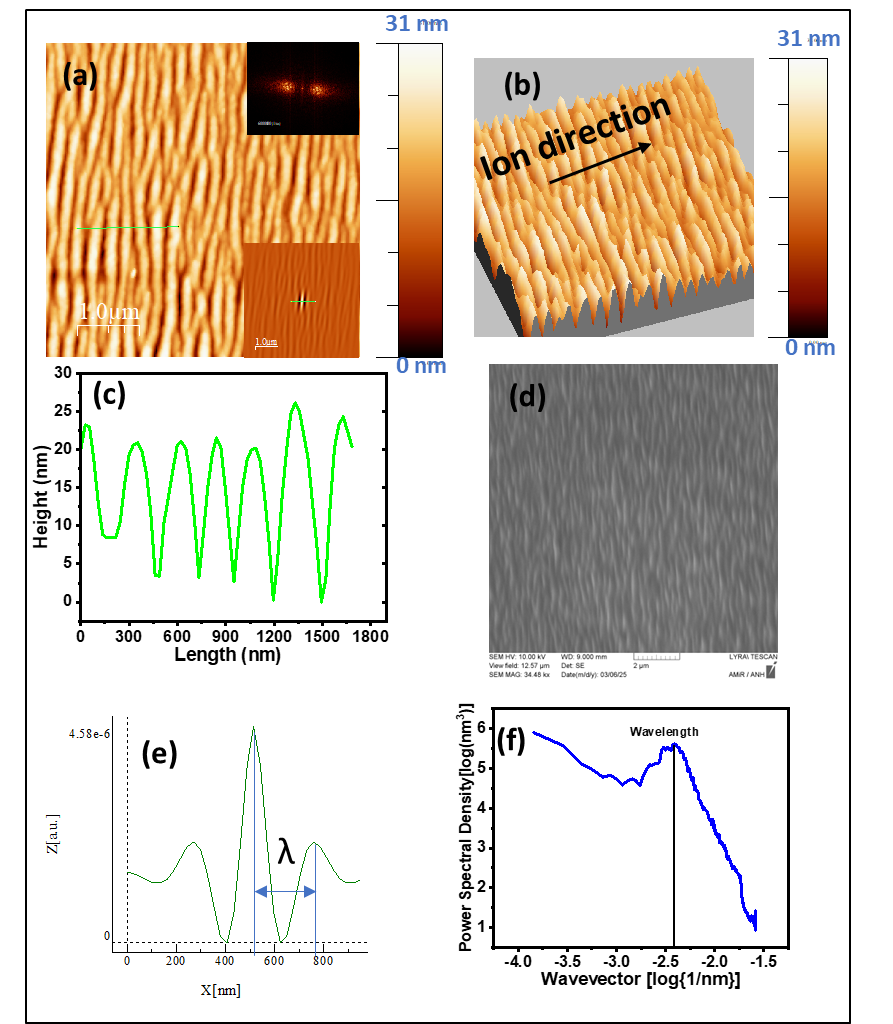


**Figure S2**: (a) Two-dimensional AFM height images showing the wavelike ripple structure at nanoscale on Si (100) surface by 30 keV Ga^+^ FIB at ion incidence angle 30^0^ with ion fluence 7×10^17^ ions/cm^2^. The inset images of right corner of (a) show fast Fourier transform (FFT) image (upper) and 2D auto-correlation image (lower), respectively. (b) 3-dimensional AFM images of ripple pattern on Si surface. The arrow on the image shows the ion beam direction, which indicates the ripple wavevector is along the ion beam direction. (c) The height profile of the ripple structure along the marked line of (a) showing the amplitude and periodicity of the ripple. (d) SEM image of the ripple structure. (e) 1-D Auto-correlation function from which the average wavelength of the ripple has been extracted as illustrated. (f) Power Spectral Density (PSD) of the ripple surface showing a clear peak corresponding to ripple wavelength. The peak in PSD also indicates the periodicity of the surface.

**S3. Nanopattern at different ion energy**


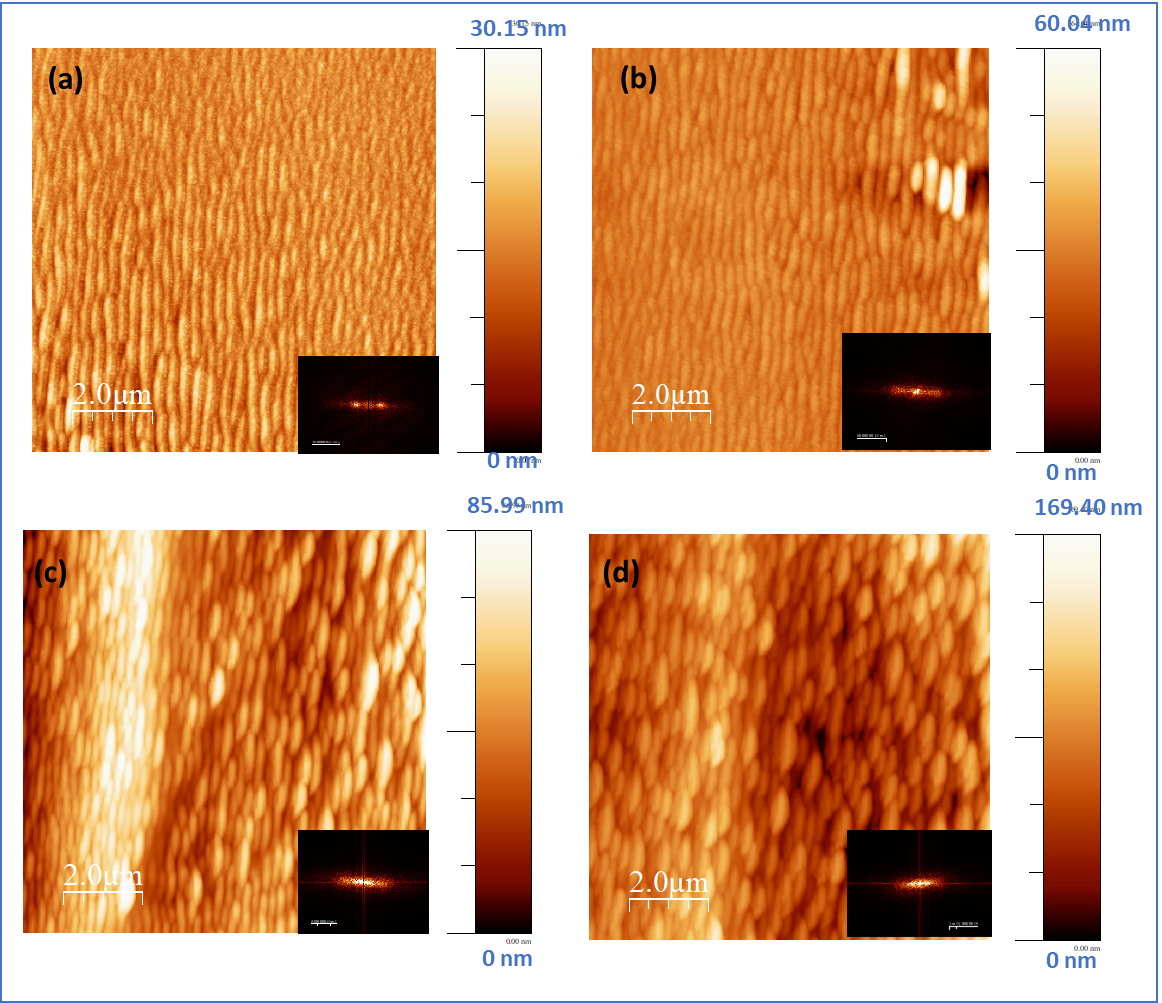
The nanopattern formation is also studied by different ion energies (10-25 keV) to observe the well-defined ripple pattern on Si surface. The AFM images of 10 keV, 15 keV, 20 keV, and 25 keV Ga^+^ FIB bombarded Si surfaces at an ion incidence angle 30^0^ with constant ion fluence 1×10^18^ ions/cm^2^ are shown in Fig. S2 (a-d). The ripple formation for all the ion energies can be observed, however, the pattern is not well-ordered as observed for 30 keV ion bombardment.

**Figure S3**: AFM height images of nano ripple patterned Si surfaces as bombarded by (a) 10 keV, (b) 15 keV, (c) 20 keV, and (d) 25 keV Ga^+^ FIB at an ion incidence angle 30^0^ with constant ion fluence 1×10^18^ ions/cm^2^. Fast Fourier Transform (FFT) images are also shown in the right corner of each AFM images.

**S4. Image of AFM probes used for Pull-off force measurement**

The pull-off force measurements are conducted by normal sharp probe and silica colloidal probe as shown in Fig. S4 (a-d). The tip apex radius is calculated from SEM and is (520±50) nm for colloidal probe and (30±10) nm for normal sharp probe. The calibration of normal and colloidal probe has been done prior to the force measurement by taking thermal noise spectra [2], details of which is reported in our earlier paper [3].


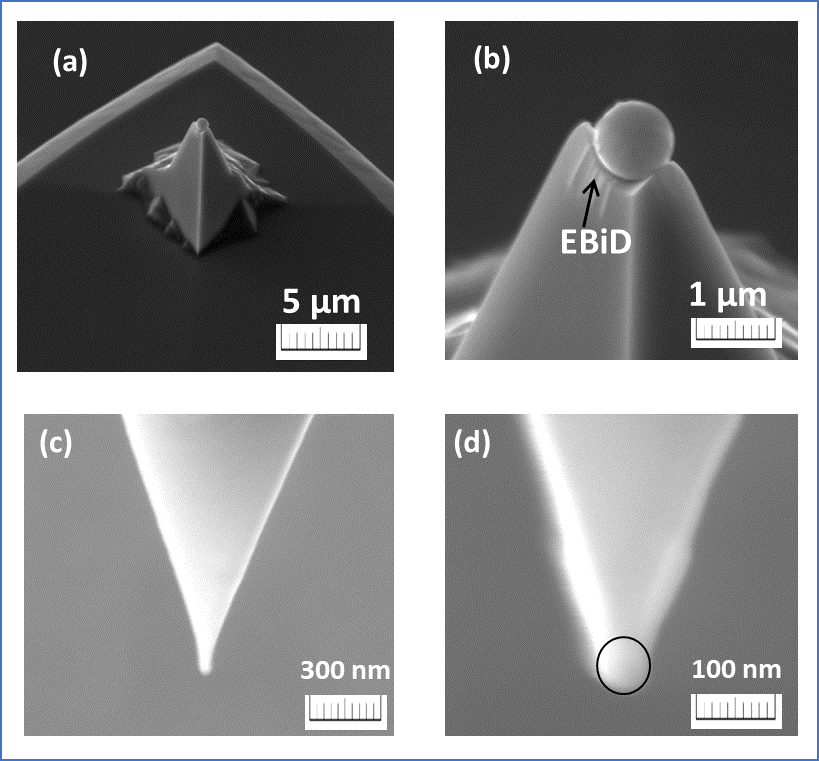


**Figure S4**: SEM images of (a&b) colloidal probe and (c&d) commercial sharp normal probe in different scale. The silica particle is attached at the end of AFM cantilever after making adhesion pocket by FIB milling and fastened by electron beam induced deposition (EBiD) as indicated in (b).

**S5. Ion penetration depth**


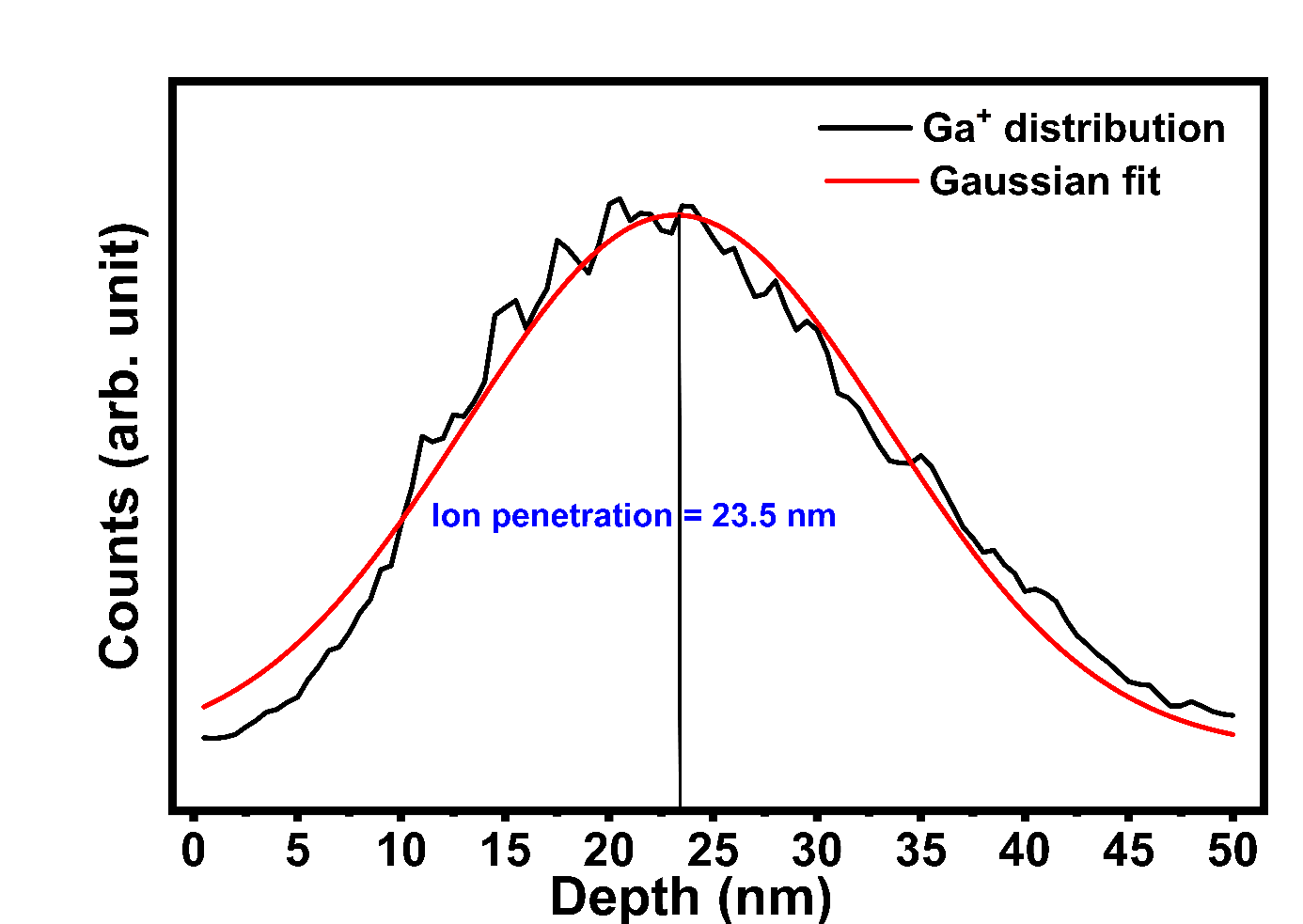
The ion penetration depth is calculated from Transport of Ions in Matter (TRIM) simulation [4] for 30 keV Ga^+^ ion on Si surface at an ion incidence angle 30^0^ as shown in Fig. S5.

**Figure S5**: Ga ion distribution profile in Si surface calculated from TRIM simulation for 30 keV ion energy at incidence angle 30^0^.

**References**

1. Horcas, I. *et al.* WSXM: A software for scanning probe microscopy and a tool for nanotechnology. *Review of Scientific Instruments* 78, (2007).

2. Hutter, J. L. & Bechhoefer, J. Calibration of atomic-force microscope tips. *Review of Scientific Instruments* 64, 1868–1873 (1993).

3. Bhowmik, D. & Fatikow, S. Probing Primary and Mechanically Degraded Nanoplastic Particles via Atomic Force Microscopy. in *2025 International Conference on Manipulation, Automation and Robotics at Small Scales (MARSS)* 1–6 (IEEE, 2025). doi:10.1109/MARSS65887.2025.11072762.

4. Ziegler, J. F., Ziegler, M. D. & Biersack, J. P. SRIM – The stopping and range of ions in matter (2010). *Nucl Instrum Methods Phys Res B* 268, 1818–1823 (2010).
